# Right ventricular-arterial uncoupling independently predicts survival in COVID-19 ARDS

**DOI:** 10.1186/s13054-020-03385-5

**Published:** 2020-11-30

**Authors:** Michele D’Alto, Alberto M. Marra, Sergio Severino, Andrea Salzano, Emanuele Romeo, Rosanna De Rosa, Francesca Maria Stagnaro, Gianpiero Pagnano, Raffaele Verde, Patrizia Murino, Andrea Farro, Giovanni Ciccarelli, Maria Vargas, Giuseppe Fiorentino, Giuseppe Servillo, Ivan Gentile, Antonio Corcione, Antonio Cittadini, Robert Naeije, Paolo Golino

**Affiliations:** 1 Department of Cardiology, Monaldi Hospital - “L. Vanvitelli” University, Naples, Italy; 2 Department of Translational Medical Sciences, “Federico II” University, Naples, Italy; 3 Department of Cardiology, Cotugno Hospital, Naples, Italy; 4 IRCCS SDN, Diagnostic and Nuclear Research Institute, Naples, Italy; 5 Department of Anesthesiology, Monaldi Hospital, Naples, Italy; 6 Department of Neurosciences, Reproductive and Odontostomatological Sciences, “Federico II” University Hospital and School of Medicine, Naples, Italy; 7 Department of Intensive Care, Monaldi Hospital, Naples, Italy; 8 Department of Clinical Medicine and Surgery, Section of Infectious Diseases, “Federico II” University Hospital and School of Medicine, Naples, Italy; 9 Department of Pathophysiology, Free University of Brussels, Brussels, Belgium

**Keywords:** COVID-19, ARDS, Right ventricular-arterial uncoupling, Echocardiography, Prognosis

## Abstract

**Aim:**

To investigate the prevalence and prognostic impact of right heart failure and right ventricular-arterial uncoupling in Corona Virus Infectious Disease 2019 (COVID-19) complicated by an Acute Respiratory Distress Syndrome (ARDS).

**Methods:**

Ninety-four consecutive patients (mean age 64 years) admitted for acute respiratory failure on COVID-19 were enrolled. Coupling of right ventricular function to the pulmonary circulation was evaluated by a comprehensive trans-thoracic echocardiography with focus on the tricuspid annular plane systolic excursion (TAPSE) to systolic pulmonary artery pressure (PASP) ratio

**Results:**

The majority of patients needed ventilatory support, which was noninvasive in 22 and invasive in 37. There were 25 deaths, all in the invasively ventilated patients. Survivors were younger (62 ± 13 vs. 68 ± 12 years, *p* = 0.033), less often overweight or usual smokers, had lower NT-proBNP and interleukin-6, and higher arterial partial pressure of oxygen (PaO_2_)/fraction of inspired O_2_ (FIO_2_) ratio (270 ± 104 vs. 117 ± 57 mmHg, *p* < 0.001). In the non-survivors, PASP was increased (42 ± 12 vs. 30 ± 7 mmHg, *p* < 0.001), while TAPSE was decreased (19 ± 4 vs. 25 ± 4 mm, *p* < 0.001). Accordingly, the TAPSE/PASP ratio was lower than in the survivors (0.51 ± 0.22 vs. 0.89 ± 0.29 mm/mmHg, *p* < 0.001). At univariate/multivariable analysis, the TAPSE/PASP (HR: 0.026; 95%CI 0.01–0.579; *p*: 0.019) and PaO_2_/FIO_2_ (HR: 0.988; 95%CI 0.988–0.998; *p*: 0.018) ratios were the only independent predictors of mortality, with ROC-determined cutoff values of 159 mmHg and 0.635 mm/mmHg, respectively.

**Conclusions:**

COVID-19 ARDS is associated with clinically relevant uncoupling of right ventricular function from the pulmonary circulation; bedside echocardiography of TAPSE/PASP adds to the prognostic relevance of PaO_2_/FIO_2_ in ARDS on COVID-19.

## Background

Severe Acute Respiratory Syndrome-CoronaVirus-2 (SARS-CoV-2) infection, or Corona Virus Infectious Disease 2019 (COVID-19), may be complicated by the acute respiratory distress syndrome (ARDS) with reported high mortality rates between 26 and 61% [1, 2]. There are data suggesting that COVID-19 respiratory failure differs from “typical” ARDS by several aspects, including preserved respiratory system compliance [3], good tolerance to hypoxemia ("*happy hypoxemia*") [4], and prominent micro- and macrovascular thrombotic changes in relation with extensive endothelial injury [5, 6]. However, whether the respiratory physiology of COVID-19-induced ARDS really differs from other types of ARDS remains discussed [7]. On the cardiac side, COVID-19 has also been associated with myocardial injury [8] and altered right ventricle (RV) strain as an independent predictor of poor prognosis [9]. There are data suggesting that COVID-19 may predominantly affect the RV and that is clinically relevant [10].

Right heart failure (“acute *cor pulmonale*”) is a long-recognized complication of ARDS, in relation to severity of the disease and ventilatory strategies associated with hyper-inflated lungs and permissive hypercapnia [11]. We hypothesized that myocardial injury and inflammatory changes in COVID-19 could be an additional cause of ARDS-related acute right heart failure. We therefore assessed the coupling of RV function to the pulmonary circulation in COVID-19 ARDS patients. To this purpose, we used bedside transthoracic echocardiography with focus on the tricuspid annular plane systolic excursion (TAPSE)/pulmonary artery systolic pressure (PASP) ratio, previously shown to be a valid surrogate of the gold standard ratio of end-systolic to arterial elastance (Ees/Ea) for the assessment of RV-arterial coupling [12] and an independent predictor of outcome in heart failure and pulmonary arterial hypertension [13].

## Methods

### Study design

This was a prospective study from two Italian centres, Ospedale dei Colli (Monaldi-Cotugno) and Federico II Hospital, Naples, Italy, which are teaching hospitals authorized for COVID-19 patients. All patients were enrolled from 8 March to 8 May 2020. The diagnosis of COVID-19 was confirmed according to the interim guidance of World Health Organization [14]. The study was approved by local Ethics Committees (#AOC/0015171/2020).

### Data collection

Patients’ demographics, clinical status, disease duration from the symptoms onset, medical history, comorbidities, laboratory examinations, concomitant treatment, type of ventilation, eventual complications, treatment, and outcomes were recorded. The diagnosis of ARDS rested on the Berlin consensus criteria and PaO_2_/FIO_2_ ratios discriminating mild, moderate, and severe forms of the disease [15]. Treatment was in keeping with current expert recommendations, with high-flow nasal O_2_ as needed to restore arterial oxygenation, and ventilation with positive end-expiratory pressure by facial mask or tracheal intubation following current expert recommendations [16]. Thus, tidal volume was kept as low as possible, on average to 6 ml/kg; positive end-expiratory pressure titrated by 2–3 cmH_2_O increments to a maximum of 10–15 cmH_2_O and a plateau pressure < 30 cmH_2_O. Noninvasive ventilation was applied when endotracheal intubation was not considered necessary.

### Transthoracic echocardiography

Bedside transthoracic echocardiographic examinations were performed with the *Vivid E9* ultrasound system (*General Electrics Medical Systems, Andover, MA, USA*), according to the American Society of Echocardiography guidelines [17]. Images were stored and analyzed offline by three independent trained observers (MD, SS and AMM).

### Statistical analysis

Kolmogorov–Smirnov test was applied to test the variable distribution. Normally distributed continuous variables were expressed as mean ± standard deviation (SD); skewed distributed continuous data were expressed as median and interquartile range [IQR]; categorical variables were expressed as counts and percentages. Two-tailed t test for paired and unpaired data was used to assess changes between groups. Linear regression analyses and partial correlation test by Pearson’s method were used to assess univariate relations. The association between analyzed variables and outcome (i.e., mortality) was established by using Cox proportional hazard regression analyses. Univariate and multivariable linear models were used to assess potential predictors of outcome. The following variables, selected according to their potential clinical relevance, were included in the analysis: age, sex, disease duration, previous lung disease, previous coronary artery disease, cardiovascular risk factors (hypertension, diabetes, obesity, smoke), therapy for COVID, type of ventilation, PaO_2_/FiO_2_ ratio, creatinine, cardiac troponin I, C-reactive protein, activated partial thromboplastin time, N-terminal pro-brain natriuretic peptide, interleukine-6, left ventricle (LV) end-diastolic diameter, LV end-systolic diameter, left atrium diameter, LV ejection fraction, mitral and aortic valve diseases, tricuspid regurgitation, TAPSE, PASP, TAPSE/PASP ratio, inferior vena cava dimension and ratio of RV to LV surface areas on an apical 4-chamber view. Results were expressed as hazard ratios with 95% confidence intervals. Outcome prediction accuracies were tested by calculating the area under the curve (AUC) for the receiver operator characteristics (ROC) curve analysis for TAPSE/PASP and PaO_2_/FiO_2_ across the endpoint. Kaplan–Meier curves for cumulative survival were constructed for the endpoint to assess the impact of TAPSE/PASP and PaO_2_/FiO_2_ on survival, categorizing patients using optimal cutoff points for TAPSE/PASP and PaO_2_/FiO_2_ derived from Youden’s Index from the ROC curve. Further, ROC curve analyses using the same multivariable model, with and without TAPSE/PASP and PaO_2_/FiO_2_, were used to investigate the gain in C-statistic for associations with outcome when compared to the same model without these parameters. Statistical analyses were performed using SPSS version 25.0 (SPSS Inc, Chicago, Illinois, USA). A value of *p* < 0.05 was considered statistically significant.

## Results

Ninety-four patients were included in the study; they presented with fever (94/94, 100%), dyspnea (87/94, 93%), fatigue (94/94, 100%) and cough (58/94, 62%). All patients had a computed tomography (CT) scan diagnostic for diffuse or localized pneumonia. The echocardiographic assessment was performed on average 3 days after hospital admission (range 1–7 days) after the patients had been stabilized with either high flow supplemental O_2_ or invasive/noninvasive ventilation.

The clinical data of the survivor and non-survivor patients are shown in Table 1.

**Table 1:** Comparison between alive and dead patients affected by COVID-19

	Alive (*n* = 69)	Dead (*n* = 25)	*p*
Age (year)	62 ± 13	68 ± 12	0.033
Sex M (%)	53 (77)	17 (68)	0.549
Disease duration (day)	7.7 ± 3.3	7.7 ± 3.1	0.942
Lung disease (%)	17 (25)	11 (44)	0.079
Coronary artery disease (%)	14 (20)	3 (12)	0.545
Cardiovascular comorbidities			
Hypertension (%)	44 (64)	19 (76)	0.362
Diabetes (%)	11 (16)	5 (20)	0.99
Smoke (%)	7 (10)	8 (32)	0.021
Obesity (%)	18 (26)	13 (52)	0.025
Treatment			
Anticoagulants (%)	69 (100)	24 (96)	0.097
Hydroxycloroquine (%)	51 (74)	19 (76)	0.840
Antivirals (%)	43 (62)	23 (92)	0.005
Monoclonal antibodies (%)	8 (12)	10 (40)	0.005
Corticosteroids (%)	14 (20)	6 (24)	0.98
Type of ventilation			
Nasal oxygen (%)	35 (51)	0 (0)	< 0.001
Noninvasive ventilation (%)	22 (32)	0 (0)	< 0.001
Intubation (%)	12 (17)	25 (100)	< 0.032
Biochemistry			
Creatinine (mg/dl)	1.3 ± 1.3	2.8 ± 1.4	< 0.001
Cardiac Troponin I (pg/l)	365 ± 644	1245 ± 2049	< 0.002
D-dimer (ng/ml)	317 ± 557	919 ± 974	< 0.001
C-reactive protein (mg/dl)	10.6 ± 19.9	22.8 ± 27.3	< 0.023
Procalcitonin (ng/ml)	0.6 ± 1.5	1.8 ± 2.0	< 0.005
APTT (sec)	36.8 ± 6.7	40.6 ± 4.3	0.037
NT-proBNP (pg/ml)	686 ± 1224	3375 ± 3891	< 0.001
Interleukine-6 (ng/ml)	33.6 ± 33.4	246.4 ± 87.4	< 0.001
PaO_2_/FiO_2_ ratio (mmHg)	270 ± 104	117 ± 56	< 0.001

Value are represented as mean ± standard deviation or absolute value and (%)

*APTT* partial thromboplastin time, *NT-proBNP* N-terminal prohormone of brain natriuretic peptide, *PaO*_*2*_ arterial partial pressure of oxygen, *FiO*_*2*_ fraction of inspired O_2_

The patient population was globally relatively old, predominantly male and presented with pulmonary comorbidities and cardiovascular risk factors. Non-survivors were older by an average of 6 years and were more frequently smokers and overweight. The majority of the patients were anticoagulated and treated with hydroxychloroquine. A proportion of the patients also received antiviral drugs, monoclonal antibodies, and corticosteroids. Non-survivors received more frequently antiviral drugs and invasive mechanical ventilation. Serum creatinine, cardiac troponin I, C-reactive protein, interleukine-6, N-terminal pro-brain natriuretic peptide, activated partial thromboplastin time, and pro-calcitonin were higher and PaO_2_/FIO_2_ lower in non-survivors. An angio CT performed when dyspnea was deemed out of proportion of standard CT imaging revealed a pulmonary embolism in nine of the patients.

Echocardiographic findings shown in Table 2 disclosed a significant increase in PASP, inferior vena cava dimensions and a decrease in TAPSE/PASP in non-survivors, as compared to survivors.

**Table 2:** Echocardiographic features

	Alive (*n* = 69)	Dead (*n* = 25)	*p*
LVEDD (mm)	48 ± 5	49 ± 4	0.388
LVESD (mm)	29 ± 7	31 ± 5	0.059
LAD (mm)	38 ± 6	40 ± 5	0.082
LVEF (%)	60 ± 7	58 ± 8	0.209
MVD	5 (7)	1 (4)	0.574
AVD	1 (1)	0 (0)	0.550
TR	2 (3)	3 (12)	0.084
TAPSE (mm)	25 ± 4	19 ± 4	< 0.001
PASP (mmHg)	30 ± 7	42 ± 12	< 0.001
TAPSE/PASP	0.89 ± 0.29	0.51 ± 0.22	< 0.001
IVC (mm)	15 ± 4	20 ± 3	< 0.001
Pericardial effusion	6 (9)	4 (16)	0.375
Echocardiographic phenotypes			
Normal	50 (73)	10 (40)	0.007
Hyperkinetic	9 (13)	3 (12)	0.99
Right	3 (4)	12 (48)	< 0.001
LV depression	3 (4)	0 (0)	0.57
Severe pericardial effusion	4 (6)	0 (0)	0.57

Value are represented as mean ± standard deviation or absolute value and (%). *p* < 0.05 statistically significant

*LVEDD* left ventricle end-diastolic diameter, *LVESD* left ventricle end-systolic diameter, *LAD* left atrium diameter, *LVEF* left ventricle ejection fraction, *MVD* mitral valve disease moderate-to-severe, *AoVD* aortic valve disease moderate-to-severe, *TR* tricuspid regurgitation, *TAPSE* tricuspid annulus plane systolic excursion, *PASP* pulmonary artery systolic pressure, *IVC* inferior vena cava

**Fig. 1:**
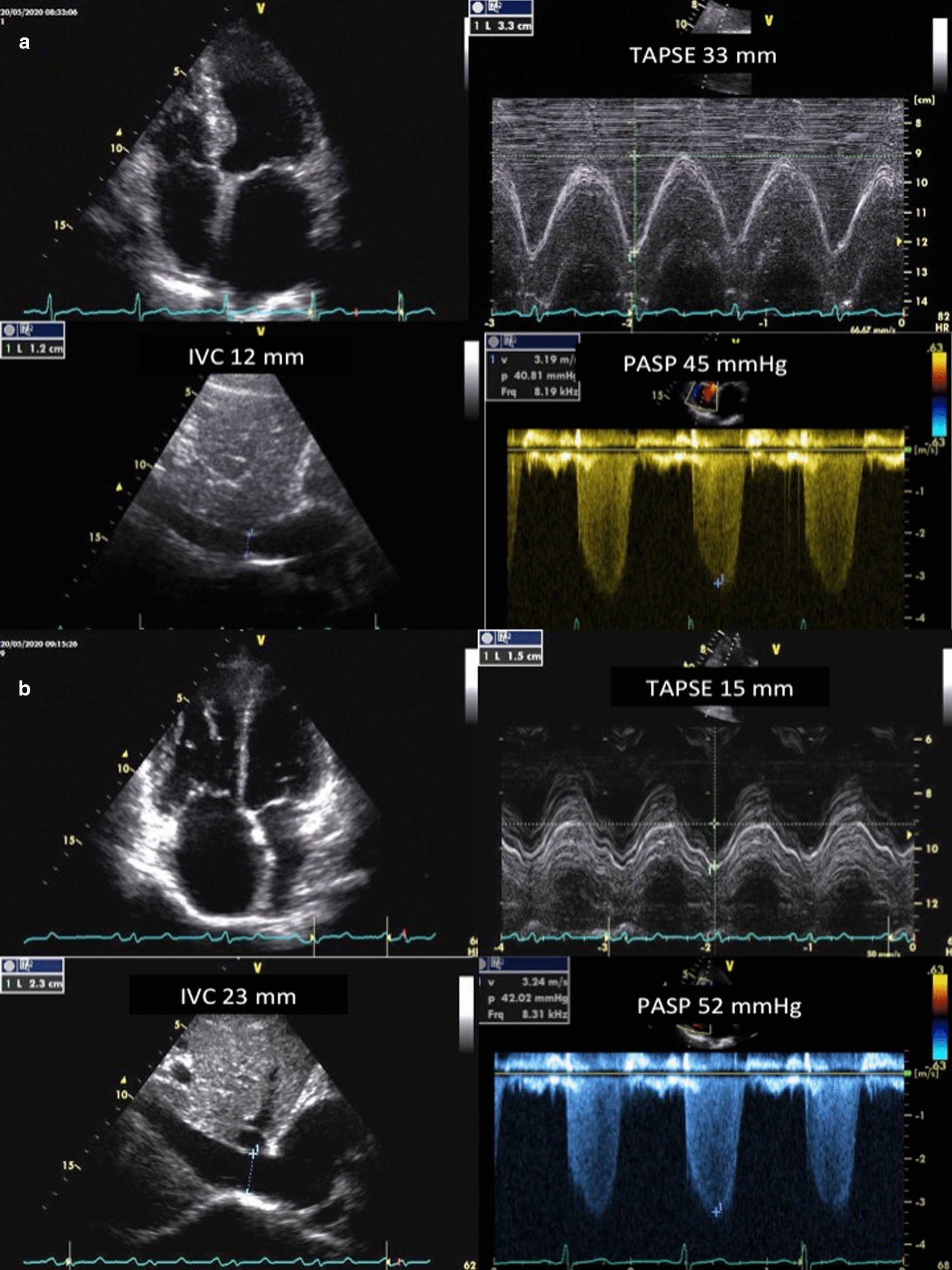
Coupling of right ventricular function to the pulmonary circulation evaluated by the tricuspid annular plane systolic excursion (TAPSE) to systolic pulmonary artery pressure (PASP) ratio. **a** Normal echocardiographic phenotype with increased pulmonary artery systolic pressure (PASP), normal tricuspid annulus plane systolic excursion (TAPSE), and preserved TAPSE/PASP. **b** Typical right heart echocardiographic phenotype with increased PASP, reduced TAPSE, low TAPSE/PASP, and right/left ventricular basal diameter ratio > 1. *IVC* inferior vena cava

A typical right heart phenotype echocardiographic examination is shown in Fig. 1 (Panel A and B).

The results of univariate and multivariable analyses are shown, respectively, in Table 3 and Table 4.

**Table 3:** Single predictor models of Cox proportional hazard analysis

Variables	HR	95% (CI)	*p*
Age (year)	1.04	1.003–1.078	0.035
Sex (female)	0.006	0.000–0.216	0.006
Fever (days)	1.001	0.912–1.098	0.98
Pulmonary disease	1.998	0.906–4.408	0.086
Coronary artery disease	0.556	0,166–1.858	0.340
Hypertension	1,767	0.706–4.429	0.224
Diabetes	1.525	0.571–4.072	0.40
Smokers	3.050	1.313–7.086	0.10
Obesity	2.252	1.027–4.936	0.044
Risk factors	–	–	0.025
0	Ref	–	–
1	6.608	0.853–51	0.07
2	5.126	0.617–42.6	0.13
3	15.518	1.861–129	0.011
4	46.105	2.772–766	0.008
Nasal oxygen	0.019	0.001–0.512	0.018
Noninvasive ventilation	0.031	0.001–1.386	0.073
Intubation	223,89	4.81–10,415	0.006
PaO_2_/FiO_2_ ratio (mmHg)	0.986	0.981–0.992	0.001
Heparin	0.274	0.037–2.039	0.206
Antivirals	5.935	1.398–25.186	0.016
Hydroxycloroquine	1.195	0.477–2.995	0.704
Monoclonal antibody	3.301	1.481–7.356	0.003
Corticosteroids	1.308	0.521–3.279	0.568
Creatinine (mg/mL)	1.236	1.067–1.432	0.005
Troponine (pg/l)	1.000	1.000–1.000	0.003
D-dimer (ng/ml)	1.000	1.000–1.001	< 0.001
C-reactive protein (mg/dl)	1.014	1.004–1.024	0.006
Procalcitonin (ng/ml)	1.108	0.975–1.259	0.115
NT-proBNP (pg/ml)	1.000	1.000–1.000	< 0.001
APTT (sec)	0.997	0.982–1.012	0.687
Interleukine-6 (ng/ml)	1.010	1.007–1.013	< 0.001
Heart rate (bpm)	1.031	1.006–1.058	0.016
Systolic blood pressure (mmHg)	0.976	0.949–1.003	0.076
Diastolic blood pressure (mmHg)	0.912	0.868–0.958	< 0.001
LVEDd (mm)	1.031	0.948–1.121	0.473
LVESd (mm)	1.039	0.99–1.091	0.123
LAD (mm)	1.066	0.995–1.143	0.068
LVEF (%)	0.972	0.934–1.011	0.151
Severe MR	0.526	0.071–3.887	0.529
Severe AR	0.049	0–201,330	0.697
Severe TR	2.671	0.798–8.95	0.111
TAPSE (mm)	0.796	0.727–0.871	< 0.001
PASP (mmHg)	1.085	1.054–1.118	< 0.001
TAPSE/PASP (mm/mmHg)	0.013	0.002–0.069	< 0.001
IVC (mm)	1.335	1.201–1.483	< 0.001
IVC respiratory changes	1.591	0.702–3.606	0.226
Pericardial effusion	1.693	0.580–4.940	0.335
Pleural effusion	0.868	0.204–3.689	0.848
Right phenotype	4.232	1.505–11.902	0.006

*APTT* partial thromboplastin time, *NT-proBNP* N-terminal prohormone of brain natriuretic peptide, *LVEDD* left ventricle end-diastolic diameter, *LVESD* left ventricle end-systolic diameter, *LAD* left atrium diameter, *LVEF* left ventricle ejection fraction, *MVD* mitral valve disease moderate-to-severe, *AoVD* aortic valve disease moderate-to-severe, *TR* tricuspid regurgitation, *TAPSE* tricuspid annulus plane systolic excursion, *PASP* pulmonary artery systolic pressure, *IVC* inferior vena cava

**Table 4:** Multivariable models of Cox proportional hazard analysis

Variables	HR	95% (CI)	*p*
Age (year)	1.002	0.944–1.063	0.953
Obesity	0.626	0.171–2.295	0.480
Creatinine (mg/mL)	1.033	0.746–1.429	0.847
Troponine (pg/L)	1.00	0.999–1.001	0.774
D-dimer (ng/mL)	1.00	0.999–1.001	0.442
C-reactive protein (mg/mL)	1.01	0.996–1.024	0.171
Heart rate (bpm)	0.996	0.961–1.032	0.817
Systolic blood pressure (mmHg)	1.038	0.988–1.09	0.137
Diastolic blood pressure (mmHg)	0.915	0.837–1.002	0.054
LVEDd (mm)	1.064	0.991–1.550	0.508
LVESd (mm)	0.899	0.707–1.143	0.385
LVEF (%)	1.022	0.900–1.161	0.739
LAD (mm)	0.947	0.858–1.046	0.947
TAPSE/PASP (mm/mmHg)	0.026	0.01–0.579	0.019
PaO_2_/FiO_2_ ratio (mmHg)	0.988	0.977–0.998	0.018

*LVEDD* left ventricle end-diastolic diameter, *LVESD* left ventricle end-systolic diameter, *LVEF* left ventricle ejection fraction, *LAD* left atrium diameter, *TAPSE* tricuspid annulus plane systolic excursion, *PASP* pulmonary artery systolic pressure, *PaO*_*2*_ arterial partial pressure of oxygen, *FiO*_*2*_ fraction of inspired O_2_

While at univariate analysis most of the biological and echocardiographic differences between survivors and non-survivors were significantly associated with survival (Table 3), only PaO_2_/FIO_2_ and TAPSE/PASP emerged as independent predictors after adjustment at multivariable analysis [hazard ratio (95% confidence interval); p value: 0.988 (0.977–0.998); *p* = 0.018 and 0.026 (0.01–0.579); *p* = 0.019, respectively] (Table 4).

**Fig. 2:**
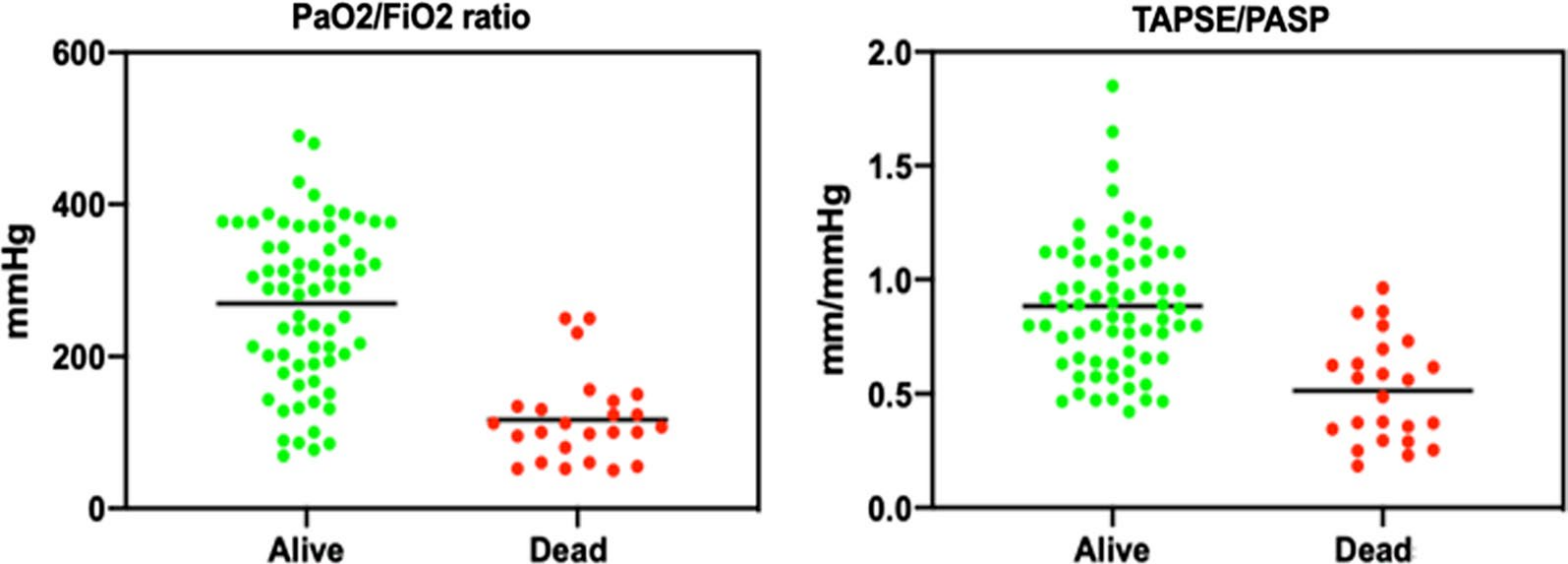
Individual values for TAPSE/PASP and PaO_2_/FIO_2_ ratios. Individual values for the tricuspid annulus plane systolic excursion (TAPSE)/pulmonary artery systolic pressure (PASP) ratio (panel **a**), and arterial partial pressure of oxygen (PaO_2_)/fraction of inspired O_2_ (FIO_2_) ratio (panel **b**). Means are indicated by horizontal bars. Both ratios were markedly decreased in non-survivors (*p* < 0.001)

**Fig. 3:**
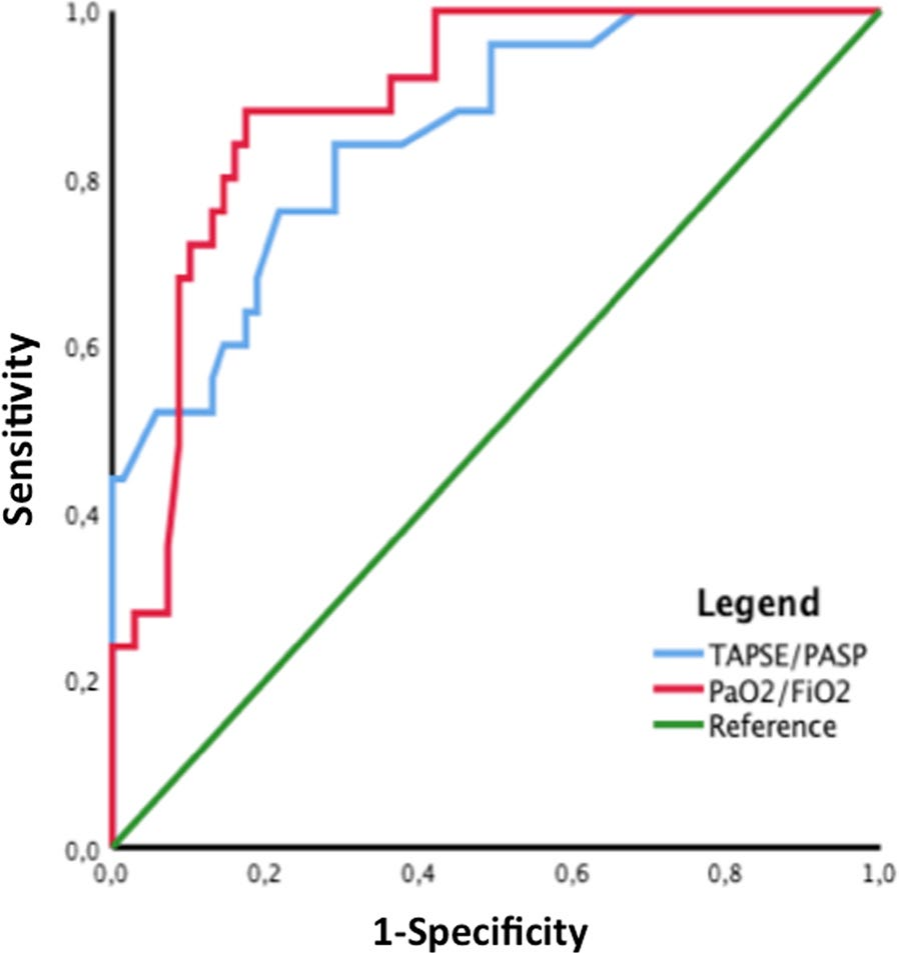
ROC curves to predict outcome of as a function of TAPSE/PASP and PaO_2_/FIO_2_. Both ratios predicted outcome with Youden indices (highest combination of sensitivity and specificity) of, respectively, 0.625 mm/mmHg and 159 mmHg. Abbreviations see Fig. 2

**Fig. 4:**
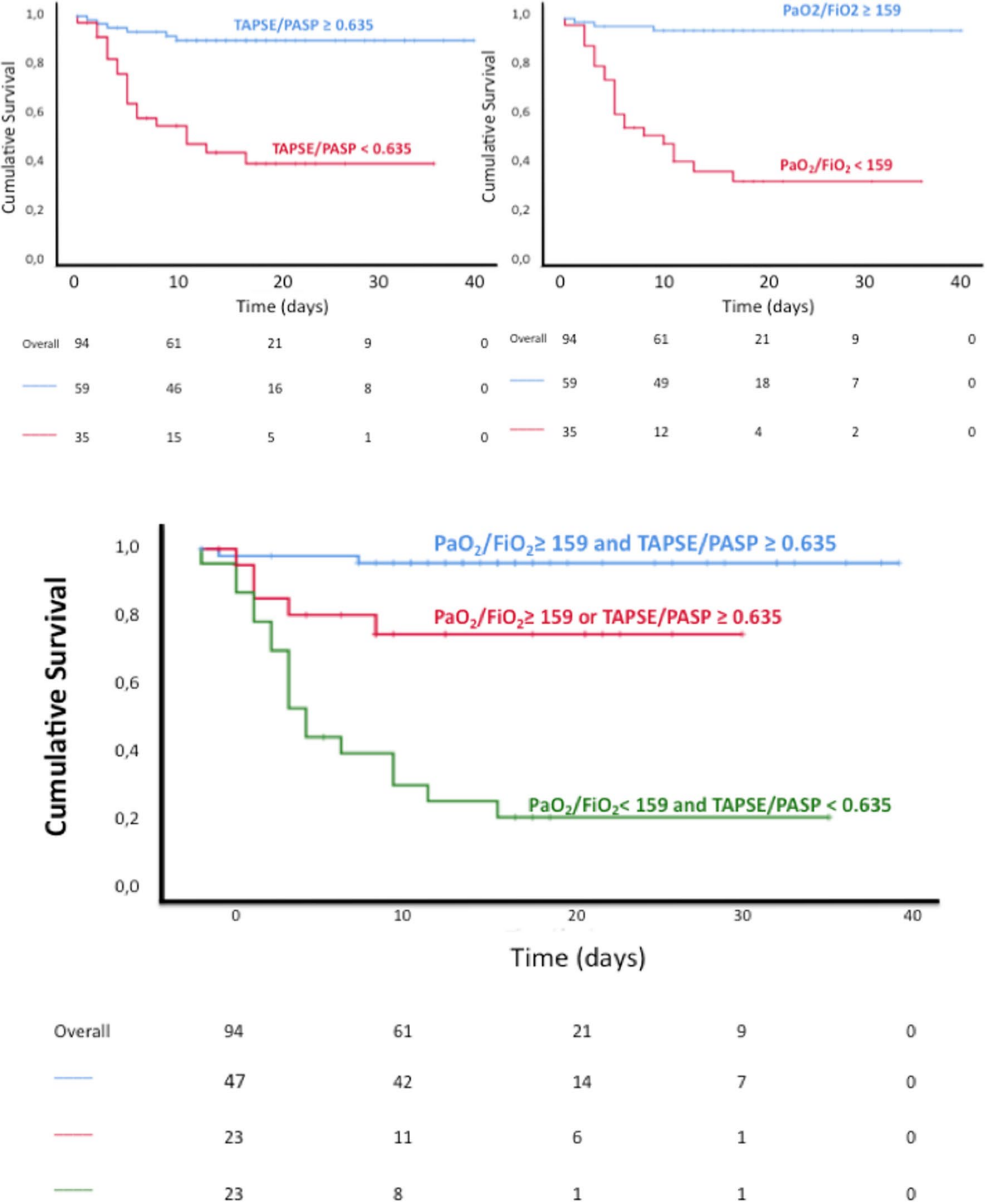
Survival according to TAPSE/PASP and PaO_2_/FIO_2_. Kaplan–Meyer curves of % survival over time as a function of TAPSE/PASP and PaO_2_/FIO_2_ above or below the ROC-determined cutoff values of 0.625 mm/mmHg and 159 mmHg, alone (upper panels) or in combination (lower panel). Abbreviations see Fig. 2

Individual values for TAPSE/PASP and PaO_2_/FIO_2_ in survivors and non-survivors are presented in Fig. 2. ROC curves to predict outcome of these two variables are shown in Fig. 3. When patients were dichotomised according to the Youden’s Index for optimal cutoff point from the ROC curve (159 mmHg and 0.635 mm/mmHg, PaO_2_/FIO_2_ and TAPSE/PASP, respectively), Kaplan–Meyer curves of % survival as a function of time in patients showed that patients with TAPSE/PASP or PaO_2_/FIO_2_ below ROC-derived cutoff values have reduced survival (chi square; log rank test *p*: 26.43; < 0.001 and 42.83; < 0.001, respectively) (Fig. 4). Furthermore, when patients were categorized according to value of both parameters, patients with reduction of both parameters showed the lowest survival (chi square: 45.87; log rank test *p*: < 0.001), significantly different to those with normal levels (chi square: 50.32, *p* < 0.001) or only one parameter impaired (chi square: 9.56, *p* = 0.001). A combination of high TAPSE/PASP or PaO_2_/FIO_2_ allowed for a very high likelihood of survival. Exclusion of the 9 pulmonary embolism patients from multivariate analysis did not affect the results.

## Discussion

The present results show that COVID 19-induced ARDS is associated with early and pronounced uncoupling of right ventricular function from the pulmonary circulation and that its noninvasive echocardiographic assessment by the TAPSE/PASP ratio adds significantly and independently to the prognostic relevance of the PaO_2_/FIO_2_ ratio in these patients.

The reported COVID-19 patients were diagnosed with pneumonia complicated by ARDS based on clinical presentation of dyspnea, cough, and fatigue; compatible chest computed tomography findings; and the PaO_2_/FIO_2_ ratio. At the moment of echocardiographic evaluation, the PaO_2_/FIO_2_ ratio had been corrected in a proportion of the survivors (Fig. 2). The patients were treated empirically with drugs expected to be of benefit, such as hydroxychloroquine and were anticoagulated. Their ventilatory management included proning, application of positive end-expiratory pressure, and so-called “protective ventilation” with a low as possible tidal volumes [16]. This resulted in a 26% mortality at the lower range of currently reported [18].

Pulmonary hypertension in the present study was mild to moderate as based on echocardiographic estimates of PASP. A PASP of 40 mmHg in the non-survivors would indeed be at the upper limit of normal taken into account age, sex, and body weight [19]. On the other hand, the TAPSE was decreased but still above the lower limit of normal in the non-survivors [20]. Accordingly, the TAPSE/PASP at 0.89 ± 0.29 in survivors was mildly decreased compared to the value of 1.11 ± 0.03 previously reported in 209 subjects older than 60 years [20]. However, it was markedly decreased to 0.51 ± 0.22 mm/mmHg in non-survivors, approaching values below 0.50 mm/mmHg previously shown to be of poor prognosis in heart failure and severe pulmonary hypertension [13].

In a recent report of 200 hospitalized with COVID-19 in non-ICU departments, PASP was > 35 mmHg in 12% and the TAPSE < 17 mm in 14.5%, but increased PASP and not decreased TAPSE was found to predict a poor outcome [21]. Mild pulmonary hypertensions along with moderate decrease in TAPSE in that study are in keeping with the present findings in more severely ill patients with respiratory insufficiency.

The TAPSE/PASP ratio was initially proposed as an estimate of RV myocardial length-tension relationship and as such showed to be of prognostic relevance in heart failure [21]. Subsequent studies confirmed its prognostic capability, not only in heart failure [22] but also in pulmonary arterial hypertension [23] and in patients with chronic lung diseases [24]. In these studies, the TAPSE/PASP was assumed to inform about RV-PA coupling, with TAPSE considered as a load-dependent surrogate of Ees and PASP as an indirect estimate of Ea [22–24]. The TAPSE/PASP has been shown to be superior to other composite echocardiographic indices in the assessment of RV-PA and correlated to gold standard invasive [12] or indirectly assessed Ees/Ea ratios [22].

As in the present study the TAPSE/PASP ratio was mostly decreased in invasively ventilated patients, one could wonder if the application of positive end-expiratory pressure could have contributed to increased PAP and RV-PA uncoupling [25]. COVID-19 ARDS patients could have presented with increased transmission of alveolar pressures to pulmonary resistive vessels because of preserved lung compliance [3]. Mechanics of the respiratory system were not assessed in the present study. However, the notion of preserved compliance in COVID-19 ARDS may not be confirmed in most of these patients [7, 26], and the “protective ventilation” approach in the present study would be expected to avoid to high volumes and alveolar pressures as a cause of excessive RV afterload [11]. This was confirmed by only mild increases in PASP disclosed by the echocardiographic examinations.

The reason for RV-PA uncoupling in the presence of only mildly increased PAP is not immediately apparent. The basic response of RV function to increased afterload is homeometric, with increased Ees (contractility) to match Ea (afterload), and uncoupling expected but only in severe or rapidly evolving pulmonary hypertension [27]. However, early RV-PA uncoupling may be observed in severe inflammatory conditions such as sepsis [28] or also in left heart failure because of negative ventricular interactions [29]. Both may occur in COVID-19 patients [8]. Therefore, the right heart in COVID-19 patients may fail even in the presence of only modest increase in afterload.

The present results are in keeping with a recent echocardiographic study in patients with COVID-19 ARDS, in which non-survivors had a PASP at the upper limit of normal, decreased indices of RV systolic function, and longitudinal strain identified as an independent predictor of outcome [9]. Pulmonary hypertension in COVID-19 may belong either to pulmonary hypertension due to lung parenchymal disease or at most probably to chronic thromboembolic pulmonary hypertension. The TAPSE/PASP is easier to assess, can be part of standard bedside echocardiographic assessments as it does not require off-line analysis of images and specific software, and may be a more sensitive assessment of RV-PA coupling. The high prevalence of RV dilatation and dysfunction in the range of 40–50% recently reported in patients with COVID-19 [10, 30] underscore the exquisite sensitivity of the RV to this newly appeared viral infection.

The most potent predictor of outcome in ARDS is the PaO_2_/FIO_2_ ratio, which as such is part of the definition of the syndrome [14]. In the present study, the TAPSE/PASP emerged with equally potent prognostic capability, suggesting a major component of acute *cor pulmonale* in COVID-19 ARDS pathophysiology. Whether this is entirely particular to COVID-19 ARDS is uncertain as there have been no systematic evaluations of RV-PA coupling in more “typical” ARDS or other viral pneumonia ARDS controls.

The present study is limited by relatively small sample size and by the small number of events. This might limit the results of the multivariate analyses and lead to a certain over-fitting. An angio-CT to diagnose acute pulmonary embolism was performed on physician in care's clinical suspicion, so that the frequency of this complication might have been under-estimated. However, PAP in the present study in the present study remained at the upper limit of normal, excluding pulmonary embolism as a cause of afterload-induced RV-PA uncoupling. Furthermore, excluding 9 of the patients with a diagnosis of pulmonary embolism did not affect the predictive capability of the TAPSE/PASP and PaO_2_/FIO_2_ ratios. Other limitations might be absence of respiratory system compliance measurements, absence of non-COVID-19 viral pneumonia controls, and exclusively noninvasive evaluations of the right heart and the pulmonary circulation. However, the results call attention to *cor pulmonale* as an important component of COVID-19 ARDS and plea for systematic bedside echocardiographic assessments added to blood gases and lung mechanics in the management of these patients.

Approximately 4 decades ago, Zapol and Snider called attention to the pulmonary circulation and the right heart in severe ARDS [31]. Pulmonary hypertension in these patients is nowadays uncommon along with progress in management, but “acute *cor pulmonale*” continues to be reported, albeit generally in the context of ventilatory settings associated with excessive increase in alveolar pressure and permissive hypercapnia [11]. The present investigation shows that acute uncoupling of the right heart from the quasi-normotensive pulmonary circulation may also occur in the context of severe systemic inflammation and vasculitis.

## Conclusions

In conclusion, COVID 19-induced ARDS is associated with early and pronounced right ventricular-arterial uncoupling, and its noninvasive echocardiographic assessment by the TAPSE/PASP ratio adds significantly and independently to the prognostic relevance of the PaO_2_/FIO_2_ ratio in these patients. These data call for the indispensable integration of bedside echocardiography in the assessment of COVID-19 patients in the intensive care setting.

## Data Availability

The data set used for this manuscript will be available from the corresponding author upon reasonable request.

## Abbreviations

ARF: Acute respiratory failure
ARDS: Acute respiratory distress syndrome
AUC: Area under the curve
COVID-19: Corona Virus Infectious Disease 2019
CT: Computed tomography
Ea: Arterial elastance
Ees: End-systolic elastance
FIO_2_: Fraction of inspired O_2_
IQR: Interquartile range
LV: Left ventricle
PaO_2_: Arterial partial pressure of oxygen
PASP: Systolic pulmonary artery pressure
ROC: Receiver operator characteristics
RV: Right ventricle
SARS-CoV-2: Severe acute respiratory syndrome-coronavirus-2
SD: Standard deviation
TAPSE: Tricuspid annular plane systolic excursion
